# Pet–Human Relationships: Dogs versus Cats

**DOI:** 10.3390/ani11092745

**Published:** 2021-09-20

**Authors:** Mónica Teresa González-Ramírez, René Landero-Hernández

**Affiliations:** School of Psychology, Facultad de Psicología, Autonomous University of Nuevo León, Nuevo León C.P. 64455, Mexico; rene.landerohr@uanl.edu.mx

**Keywords:** human–animal interactions, dog–owner relationship, cat–owner relationship

## Abstract

**Simple Summary:**

Human–animal interactions have been the focus of research in recent decades, with the primary interest being the dog–owner relationship. The cat–owner relationship has not been as well studied, nor has the comparison between the two types of relationships. To compare these relationships, 132 people residing in Mexico who lived with both dogs and cats were evaluated. The results of the study indicate that the perceived relationship that an individual has with cats is better than that with dogs because the perceived cost of the relationship is greater with dogs and the interaction is greater with cats. However, the emotional closeness that occurs in the relationship is greater with dogs.

**Abstract:**

The study of human–animal interactions has increased, focusing on the dog–owner relationship, leaving a lag in research on the cat–owner relationship and practically a total absence of studies that compare the dog–owner relationship with the cat-owner relationship. Therefore, the objective of the present study was to make this comparison based on the perception of people living with both dogs and cats, considering interaction, emotional closeness, and perceived cost of the relationship. A total of 132 residents in Mexico participated. To evaluate the pet–human relationship, the dog and cat versions of the Monash dog owner relationship scale were used, thus obtaining comparable scores for the relationship with dogs and cats. Based on what the owners reported, significant differences were found. Relationships with cats were better than relationships with dogs, a finding that was confirmed when comparing male dogs and cats and when comparing female dogs and cats. It was concluded that relationships with cats are better because the perceived cost of such a relationship is lower. However, emotional closeness is greater with dogs than with cats.

## 1. Introduction

In recent decades, interest in studying human–animal interactions has increased [1], and the results of such studies has revealed that owners of dogs and cats tend to have better physical health than non-owners or owners of other types of pets [2], fewer visits to the doctor than people without pets [3], less loneliness [4], and a decrease in depressive symptoms and negative feelings and an increase in self-esteem and social interactions [5], among other benefits documented in review studies [6,7]. Nevertheless, other researches have shown no differences between dog owners and non-dog owners in self-reported mental health, general health, loneliness, the frequency of social contact [8], life satisfaction [9] and happiness [9,10].

The benefits of pet ownership are not guaranteed and might depend on many different factors such as the appropriateness of a particular dog as a pet [11] or how an individual perceives the relationship with their dog. The greatest benefits in perceived health and stress levels appear to occur when the dog is regarded as a family member [12].

Many of these studies have focused on human–dog interactions. In 2007 [13], it was documented that studies regarding the human–cat bond were not as frequent, a finding that continues to this day, and there are still aspects regarding cats and the cat–owner relationship that require more research [13]. Thus, cats have not played an important role in research, and few studies have examined the favorable attributes or positive benefits of cat ownership; in the studies in which cats have been included, fewer positive effects or more contradictory results have been reported than in studies in which dogs are included [13]. For example, compared to having a cat, having a dog has been associated with greater well-being [14]; however, it has also been found that the relationship with dogs and cats is perceived as equally beneficial, although people who have dogs seem to benefit more by exercising and appreciating nature [15].

There are different scales to evaluate the owner–pet relationship. Although there are more than 100 scales that evaluate human–animal interaction [16], the Monash dog owner relationship scale (MDORS) is the most robust scale to measure quality of dog–owner relationship, from the owner’s perception [17]. The Monash dog owner relationship scale (MDORS) [18] was developed from social exchange theory, which specifies that relationships are maintained only when the perceived cost and benefits are balanced or when the perceived benefits are greater than the costs of the relationship. Thus, the scale is composed of three subscales that evaluate perceived emotional closeness, which refers to perceived social support, bonding, companionship and unconditional love. Interaction refers to the activities related to the care of a dog such as grooming, but also to behaviors such as kissing or hugging the dog and the time and the emotional experiences that are shared. Perceived cost refers to the cost of caring for a dog including economic aspects, the increase in responsibility, and the restrictions for the owner [18]. Recently, the MDORS was adapted to evaluate the cat–owner relationship [19] (i.e., the cat–owner relationship scale (CORS)), preserving the three subscales described. To use a measure that permits the comparison between the relationship with dogs and with cats was another reason to select MDORS and CORS for use in this study.

According to theory [18], for a relationship to be maintained, there must be a balance between the aspects evaluated by the MDORS/CORS. The satisfaction of being a dog caregiver has been associated with the MDORS score, and the high perceived cost reduces the probability of being satisfied with the relationship [20].

A healthy relationship will benefit humans [11,12] and will motivate them to ensure a good quality of life for companion animals. A consequence of a broken dog–owner bond due to canine behavior problems is the possibility that a dog could be abandoned or euthanized [21]; more than 30% of abandoned dogs were abandoned due to behavioral problems [22]. Thus, a poor relationship can lead to negligent care of the companion animal or even its abandonment [19].

For cats, although they form a close emotional relationship with humans, little is known about this relationship; the type of relationship formed is the product of the dynamics existing between the human and the cat; having a greater understanding of this relationship leads to the better provision of care to cats, improves the relationship between a cat and its owner, and deepens the potential benefits of having a cat [23].

Studies focused on identifying the characteristics of the most successful owner–animal relationships will benefit the study of well-being for humans and animals involved in the relationship [15]. Considering the above and the fact that characteristics influencing the dog–owner relationship have long been studied [24], with no studies examining the dog–owner vs. cat–owner relationship, the present study aims to compare the dog–owner relationship with the cat–owner relationship based on the perceptions of people living with both dogs and cats, considering the three aspects included in the MDORS/CORS: interaction, emotional closeness, and perceived cost.

## 2. Materials and Methods

The methodology and ethical aspects of this study were approved by researchers from the Research Group focused on Social and Health Psychology of the Universidad Autónoma de Nuevo León (Autonomous University of Nuevo León), the number is CAPS-20-19-11.

### 2.1. Participants

Owners of both dogs and cats living in Mexico participated in the study. Snowball sampling was used, which asks participants to ask another person who had a dog and a cat to answer the questionnaire. An online system (SurveyMonkey.com) was used. The survey link was posted on the author’s wall on Facebook, and contacts were asked to share it. Role in pet care was not asked, thus, each participant may or not be the primary caregiver of the animal. No characteristics of companion animals were used as inclusion criteria. Incomplete questionnaires were discarded. In total, 132 people who had at least one dog and one cat as pets participated in this study. The mean age of owners was 35.6 years (SD = 11.9); 86.4% were women (*n* = 114), and 13.6% were men (*n* = 18). Of these, 59.1% were single, 36.4% were married or in common law relationships, 2.3% were divorced or separated, and 2.3% were widowed. The characteristics of the dogs and cats are described in the Results section.

### 2.2. Instruments

To evaluate the pet–owner relationship, the dog and cat versions of the MDORS were used, thus allowing the scores for the dog–owner and cat–owner relationships to be compared.

To evaluate the dog–owner relationship, we applied the Monash dog owner relationship scale [18] translated to Spanish, using the back translation method for the Mexican population (MDORS-M) [25]. This scale is considered a robust instrument for the evaluation of the human–dog relationship, from the perception of the human [17]. The scale has 28 items arranged in three subscales (i.e., (1) dog–owner interaction; (2) perceived emotional closeness; and (3) perceived costs) scored using a Likert scale, with options ranging from 1 to 5. For scoring, the scale for perceived costs was reversed, and the scores were added to those for the items on the other two subscales. The resultant score indicates the strength of the relationship based on the perception of the owner. In its Mexican adaptation, the MDORS-M scale presented a reliability of α = 0.82 for the dog–owner interaction subscale; α = 0.91 for the emotional closeness subscale; and α = 0.81 for the perceived costs subscale. This scale presents an adequate general reliability of α = 0.88 [25].

To evaluate the cat–owner relationship, the CORS was used, adapted by Howell et al. [19] from the MDORS [18], in its Spanish version for Mexico validated by González-Ramírez and Landero-Hernández [26]. Spanish translation of MDORS was used for CORS adaptation, replacing the word dog with cat and using the back translation method for the items added by Howell et al. for the CORS [18]. The CORS consists of 26 items scored using a 5-point Likert-type scale. The CORS is divided into three subscales, with six items on the cat–owner interactions subscale, 11 items on perceived emotional closeness subscale, and nine items on the perceived costs subscale. Howell et al. [19] reported adequate psychometric properties for CORS. In the version for Mexico, the internal consistency was 0.84; for the closeness subscale, the alpha was 0.86; for the interaction subscale, the alpha was 0.80; and for the perceived cost subscale, the alpha was 0.74 [26].

For both scales, a higher score indicates a stronger presence of the variable; that is, a higher score reflects a higher perceived cost, greater emotional closeness, and a better interaction. For the sum of the MDORS/CORS scores, the items for perceived cost were recoded. The mean scores, calculated by dividing the total score by the number of items, are presented. Thus, although the number of items differs between the MDORS and the CORS, the scores are comparable using mean scores. The Cronbach alpha coefficients for both scales for this study are presented in the results.

### 2.3. Procedure

Snowball sampling was used, which asked participants to seek for people who had a dog and a cat to answer the questionnaire. An online system (SurveyMonkey.com (accessed on 10 July 2019)) was used. The survey link was posted on the first author’s wall on Facebook, and contacts were asked to share it. Role in pet care was not asked, thus, each participant may or not be the primary caregiver of the animal. Participants were instructed to choose one of their cats and one of their dogs if they had more than one when providing demographic information (age and sex) and completing the questionnaires. Questionnaires were responded in the same order by all participants; first responded regarding their cat and then about their dog.

### 2.4. Statistical Analysis

To evaluate the difference between the dog–owner vs. cat–owner relationship scale scores, the Wilcoxon signed-rank statistical test was used; because they are paired groups, the same person answered each questionnaire regarding their dog and cat. The Mann–Whitney U test was used for pet sex comparisons, and Spearman correlation was used to analyze relationships with age. Nonparametric tests were used because the scores did not fit a normal distribution when evaluated with the Kolmogorov–Smirnov test (*p* < 0.05).

## 3. Results

The participants indicated having a mean of 2.5 cats (SD = 2.7, median = 2.0) and 1.8 dogs (SD = 1.0, median = 1.5). The mean age of the cats was 4.7 years (SD = 3.7); 52.3% of the cats were female, and 47.7% were male. The mean age of the dogs was 5.6 years (SD = 4.4); 54.5% of the dogs were female, and 45.5% were male.

Based on the information reported by the owners, a significant difference was found in pet–owner relationships, both in the total and subscale scores for the CORS/MDORS. The owners reported greater interaction and lower perceived cost with their cats and greater emotional closeness with their dogs. Likewise, the total score indicates that relationships with cats are better than relationships with dogs (Table 1). This finding was confirmed when comparing male dogs and cats and when comparing female dogs and cats (Table 2).

The cat–owner relationship was better with male cats; specifically, greater emotional closeness and lower perceived cost were reported. For dogs, the only significant difference was in emotional closeness, with a higher score for male dogs (Table 3).

When evaluating the correlation with the age of the companion animal, the only subscale with a significant and negative correlation was perceived costs, both for cats (r_s_ = −0.263; *p* = 0.002) and dogs (r_s_ = −0.349; *p* = 0.001), indicating that young companion animals imply a higher perceived cost for the owners.

## 4. Discussion

The main purpose of this study was to compare the dog–owner relationship with the cat–owner relationship, based on the perception of people who lived with both dogs and cats. In the only similar study that we came across, it was found that humans perceive the relationship with dogs and cats as beneficial in equal measure, although it is reported that dogs help their owners exercise and allow them to appreciate nature more, likely due to the demand for exercising dogs and the consequent need for going outside for walks with them [15].

The scores found in this study indicated greater emotional closeness and less interaction with dogs than those reported in the study by Meyer and Forkman [24] and higher perceived cost than that reported in a study of González-Ramírez et al. [25]. Regarding the scores for cats, the results did not differ from those reported by González-Ramírez and Landero-Hernández [26].

The participants indicated greater emotional closeness with their dogs than with their cats, indicating that people perceived greater social support, companionship, and unconditional love [18] with their dogs. However, the scores for cats were higher for interaction and lower for perceived cost, indicating that participants spent more time stroking, brushing, and hugging their cats than their dogs and, in turn, felt that the relationship with their cats was less expensive, required less responsibility, and involved less restrictions in their (owners’) daily activities [18]. Thus, based on the balance between the benefits and costs of the relationship, as indicated in social exchange theory, which suggests that the relationship overall was better with cats. In addition, the findings were consistent with data that indicate that perceived costs change the probability that a person will feel satisfied with the relationship with their dog [20].

These results explain in part why the number of households that prefer cats as pets has increased recently [19] and that, in some countries, the number of cats has exceeded that of dogs as pets [13]; and in Europe, they are the most common pet [27]. Although in Mexico, dogs are still the most common pet, there has not been an exclusive census of pets in Mexico. With data from the population census, it is estimated that 57% of households own a pet; 85% of them own a dog; and 15% own a cat [28,29]. There is no information regarding other pets. From 2008 to 2018, an increase of 20% in households with a dog was reported [28,29]. Regarding cats, in 2015, the Mexican Association of Cat Medicine reported that the trend of keeping cats as pets was increasing as people considered that they did not need as much attention as dogs, could live in small spaces, are independent, long-lived, and clean [30].

When analyzing the data, it became apparent that comparing the scores based on the sex of the pets would be wise. Significant differences were found in all comparisons (female dog vs. female cat; male dog vs. male cat), confirming a better relationship with both male and female cats and only a higher score in emotional closeness for both male and female dogs.

Comparing male cats with female cats, participants reported a better relationship with males, with whom they had greater emotional closeness and less perceived cost in the relationship; however, there were no differences in interactions with male cats and female cats. With dogs, the only significant difference was in emotional closeness, with a higher score for male dogs.

These analyses resulted in another original contribution of the study because no previous studies were found that presented this comparison by applying MDORS or CORS. However, we did find a study in which the sex of dogs did not explain the variance in MDORS scores [24]. In another study that utilized the MDORS, which sought to identify characteristics that would explain whether the relationship was classified as a higher emotional dog–owner bond or a lower emotional dog–owner bond, none of the characteristics of dogs including age and sex functioned as an explanatory variable [31].

Another relevant aspect is that the participants felt, for both dogs and cats, that the cost of the relationship was higher when the pets were younger, a finding that is explained by the highest-scoring questions referring to pets making a mess and to a substantial amount of money being spent on pets. It is important to consider that in this sample, few dogs and cats were geriatric, no dog was older than nine years old, and only 10% of cats were 10 years old or older. In another sample, a higher perceived cost could be found in older pets, which was associated with the economic costs due to deterioration in health and behavioral changes.

It is necessary to continue with studies on the relationships between owners and their dogs and cats, and these investigations should aim to clarify the characteristics of the most successful relationships and the benefits for both parties of the relationship [15,32].

Among the limitations of the study is the fact that most of the participants were women: only 18 men responded. Female bias in survey and questionnaire participation is widely reported in previous studies that have investigated the relationship with dogs or cats [15,23].

Another limitation is the lack of information regarding whether pets spend most of their day indoors or outdoors. In an international study, the results indicated that the number of indoor cats is likely to increase with increasing urbanization and that the main reason for keeping cats indoors, regardless of the country, was concern about traffic. Although Mexico was not included in the aforementioned study, this reason for keeping cats indoors would be applicable to the country [33]. For dogs, it is still common in Mexico for dogs to spend most of their time in the courtyard or garage of the house. Thus, it is possible that dogs spend less time indoors than cats, which would explain the higher score in cat–owner interaction subscale.

Future studies should include questions that can help identify where dogs and cats spend most of their time (indoors/outdoors), which could explain the results found. Knowing MDORS and CORS differences between neuter/spay status is likely to be important as well as evaluating the relationship between dogs and cats. In a study by Feuerstein and Terkel [34], it was found that the relationship between the two species was amicable. Comparing the MDORS and CORS scores based on whether the relationship between dogs and cats is amicable or not will provide more elements to explain the pet–owner relationship and, as above-mentioned, the benefits of this relationship for both humans as well as for dogs and cats.

## 5. Conclusions

Based on the results obtained, it was concluded that for this sample of participants residing in Mexico, their relationship with cats was better than the relationship with their dogs, due in large part to the fact that the perceived cost of the relationship with cats is less. Emotional closeness was greater with dogs than with cats.

## Figures and Tables

**Table 1:** Differences between the relationship with dogs and cats.

Variable	Cat Me	Cat M	Cat SD	Alpha	Dog Me	Dog M	Dog SD	Alpha	Wilcoxon Rank Test
Pet–owner interaction	4.8	4.6	0.6	0.81	3.4	3.3	0.8	0.85	Z = −9.300; *p* = 0.001
Perceived emotionalcloseness	3.9	3.9	0.7	0.88	4.5	4.3	0.9	0.94	Z = −6.522; *p* = 0.001
Perceived costs	1.7	1.7	0.5	0.68	1.9	2.2	0.8	0.86	Z = −6.059; *p* = 0.001
CORS/MDORS	4.2	4.2	0.5	0.88	3.9	3.8	0.6	0.92	Z = −6.801; *p* = 0.001

Me: Median; M: Mean; SD: Standard deviation.

**Table 2:** Differences between the relationship with dogs and cats, by pet sex.

Females	Female Cat			Female Dog			Mann-Whitney U
	Me	M	SD	Me	M	SD	
Pet–owner interaction	4.8	4.5	0.7	3.4	3.2	3.4	Z = −7.948; *p* = 0.001
Perceived emotional closeness	3.8	3.8	0.7	4.4	4.2	4.4	Z = −4.458; *p* = 0.001
Perceived costs	1.8	1.9	0.4	1.9	2.1	1.9	Z = −2.208; *p* = 0.027
CORS/MDORS	4.1	4.1	0.5	3.8	3.8	3.8	Z = −3.492; *p* = 0.001
**Males**	**Male Cat**			**Male Dog**			**Mann-Whitney U**
	**Me**	**M**	**SD**	**Me**	**M**	**SD**	
Pet–owner interaction	4.8	4.7	0.4	3.5	3.4	0.8	Z = −8.705; *p* = 0.001
Perceived emotional closeness	4.5	4.1	0.7	5.0	4.3	0.9	Z = −3.474; *p* = 0.001
Perceived costs	1.4	1.7	0.5	2.1	2.3	0.8	Z = −4.523; *p* = 0.001
CORS/MDORS	4.4	4.3	0.4	4.1	3.8	0.8	Z = −3.830; *p* = 0.001

Me: Median; M: Mean; SD: Standard deviation.

**Table 3:** Intragroup differences by pet sex.

Cats	Female Me	Female M	Female SD	Male Me	Male M	Male SD	Mann-Whitney U
Pet–owner interaction	4.8	4.5	0.7	4.8	4.7	0.4	Z = −1.359; *p* = 0.174
Perceived emotionalcloseness	3.8	3.8	0.7	4.5	4.1	0.7	Z = −2.609; *p* = 0.009
Perceived costs	1.8	1.9	0.4	1.4	1.7	0.5	Z = −2.843; *p* = 0.004
CORS	4.1	4.1	0.5	4.4	4.3	0.4	Z = −3.100; *p* = 0.002
**Dogs**							
Pet–owner interaction	3.4	3.2	0.8	3.5	3.4	0.8	Z = −1.237; *p* = 0.216
Perceived emotionalcloseness	4.4	4.2	0.8	5.0	4.3	0.9	Z = −2.720; *p* = 0.007
Perceived costs	1.9	2.1	0.7	2.1	2.3	0.8	Z = −0.660; *p* = 0.509
MDORS	3.8	3.8	0.5	4.1	3.8	0.8	Z = −1.689; *p* = 0.091

Me: Median; M: Mean; SD: Standard deviation.

## Data Availability

The data that support the findings of this study are available on request from the corresponding author.
